# Development of COVID-19 vaccine using a dual Toll-like receptor ligand liposome adjuvant

**DOI:** 10.1038/s41541-021-00399-0

**Published:** 2021-11-18

**Authors:** Mayuresh M. Abhyankar, Barbara J. Mann, Jeffrey M. Sturek, Savannah Brovero, G. Brett Moreau, Anjali Sengar, Crystal M. Richardson, Sayeh Agah, Anna Pomés, Peter M. Kasson, Mark A. Tomai, Christopher B. Fox, William A. Petri

**Affiliations:** 1 Department of Medicine, Division of Infectious Diseases and International Health, University of Virginia, Charlottesville, VA 22903 USA; 2 Department of Medicine, Division of Pulmonary and Critical Care Medicine, University of Virginia, Charlottesville, VA 22903 USA; 3 Departments of Molecular Physiology and Biomedical Engineering, University of Virginia, Charlottesville, VA USA; 4 INDOOR Biotechnologies, Inc., Charlottesville, VA 22903 USA; 5 3M Corporate Research and Materials Lab, 3M Center, 270-4N-04, St Paul, MN 55144 USA; 6 Infectious Disease Research Institute, Seattle, WA 98102 USA

**Keywords:** Adjuvants, Adaptive immunity

## Abstract

We developed a SARS-CoV-2 spike subunit vaccine formulation containing dual TLR ligand liposome adjuvant. The vaccine-induced robust systemic neutralizing antibodies and completely protected mice from a lethal challenge. Two immunizations protected against lung injury and cleared the virus from lungs upon challenge. The adjuvanted vaccine also elicited systemic and local anti-Spike IgA which can be an important feature for a COVID-19 vaccine.

## Introduction

Respiratory tract infections remain the top cause of morbidity and mortality from infectious diseases worldwide^1^. The explosive outbreak of severe acute respiratory syndrome coronavirus 2 (SARS-CoV-2) that led to the COVID-19 pandemic underlines a persistent threat of respiratory tract infectious diseases and warrants preparedness for a rapid response. COVID-19 is primarily a lower respiratory tract infection that can result in flu or pneumonia-like symptoms. Severe disease can manifest as an acute respiratory distress syndrome (ARDS), multi-organ failure, cytokine storm, or death^2^. Numerous studies characterizing the immune response in COVID-19 patients have revealed more disparities than commonalities^3–6^. At the time of writing, at least 108 vaccines based on different technologies were being assessed in various phases of clinical development and twenty vaccines had been authorized for emergency use worldwide by various regulatory authorities^7^. The emergence of more transmissible variants, unavailability of data on the longevity of response, the effect of age, and co-morbidities on response to the vaccine are additional knowledge gaps that need to be addressed. It is therefore imperative to explore various vaccine platforms and strategies in parallel^8^. SARS-CoV-2 is a single-stranded positive-strand RNA virus and spike protein is the primary target of neutralizing antibodies^9^. An effective vaccine should induce high titers of neutralizing antibodies preferably with minimum antigen amount and doses. Incorporating a suitable adjuvant in a vaccine may address these requirements. Arunachalam *et al* recently evaluated the potential of adjuvanted SARS-CoV-2 spike protein receptor-binding domain (RBD) to elicit the neutralizing response in monkeys. Although all the five adjuvants tested induced substantial neutralizing antibody titers, different profiles of Th1-Th2 responses as well as varying levels of protection against SARS-CoV-2 were observed depending upon the adjuvant platform^10^. Toll-like receptors (TLRs) are a category of pattern-recognition receptors critical for pathogen recognition. TLR agonists have been extensively studied as vaccine adjuvants as they allow rapid activation of innate immunity, and subsequently, effective adaptive immunity^11^. We have recently developed a fully synthetic dual TLR nanoliposome adjuvant that can concurrently elicit antigen-specific mucosal and systemic responses^12,13^. The TLR4 agonist GLA favored a mucosal IgA response whereas TLR 7/8 agonist 3M-052 elicited a robust systemic Th1 response. This adjuvant can be used for mucosal immunization, and an intranasal route of administration was found to be important for the generation of these immune responses^13^. Based on these observations, we tested the protective capability of adjuvanted Spike vaccine using the SARS-CoV-2 K18-hACE2 mouse infection model^14^.

## Results

### Adjuvanted spike vaccine protected against lethal infection

**Fig. 1:**
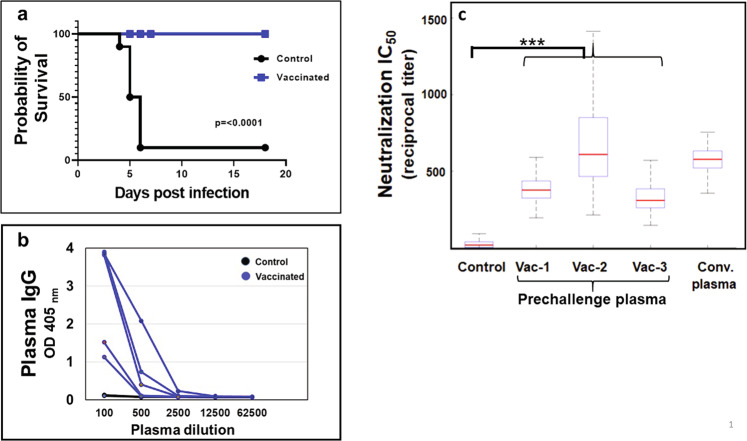
Adjuvanted Spike vaccine protected against SARS-CoV-2 lethal challenge and elicited neutralizing antibody response. K18-hACE2 mice (*n* = 10 per group) were immunized on days 0, 14, and 28 with adjuvanted full-length spike protein using a combination of subcutaneous (SC, days 0 and 28) and intranasal (IN, day 14) routes. Mice were challenged with SARS-CoV-2 two weeks after the final immunization. a Survival analysis: Nine out of ten control mice succumbed to infection by day-6 postchallenge. All the vaccinated mice survived until the termination of the experiment (day-18). **b** Anti-S1 IgG: Prechallenge plasma was collected a week after final immunization and anti-S1 IgG levels were measured by ELISA. **c** Pseudovirus entry neutralization. Prechallenge plasma obtained a week post-third immunization was evaluated to assess the ability to inhibit pseudovirus entry in Vero E6 cells. Plasma from immunized mice (Vac-1 to Vac-3) showed neutralization capability equivalent to human convalescent plasma (conv. plasma). Boxes denote mean with standard deviation. Asterisk indicates that the results are statistically significant as follows: ***p < 0.001.

The protective efficacy of GLA 3M-052–liposomes adjuvanted full-length spike antigen was evaluated in a mouse model of SARS-CoV-2 infection^14^. K18-hACE2 mice, (*n* = 10 per group), were immunized three times with adjuvanted spike vaccine using a mixed regimen comprising of a subcutaneous prime followed by an intranasal and a subcutaneous boost. A 2-week interval was maintained between each immunization^13^. The antigen and adjuvant doses for the immunizations were based on the design of experiments statistical approach^15^. Mice in the control group received only adjuvant. All the mice were challenged 2 weeks after the third immunization intranasally with 1300 pfu SARS-CoV-2. Nine of ten control group mice succumbed to infection by day-6 postchallenge, whereas all the mice from the vaccinated group survived (Fig. 1a) and displayed no clinical signs for the duration of the experiment (19 days postchallenge). Prechallenge plasma collected a week after final immunization showed high anti-Spike IgG titer (Fig. 1b) that effectively neutralized entry of spike pseudotyped virus into Vero cells with an IC_50_ value that was equivalent to convalescent plasma from COVID-19 patient (Fig. 1c).

### Two immunizations protected against lung immunopathology

**Fig. 2:**
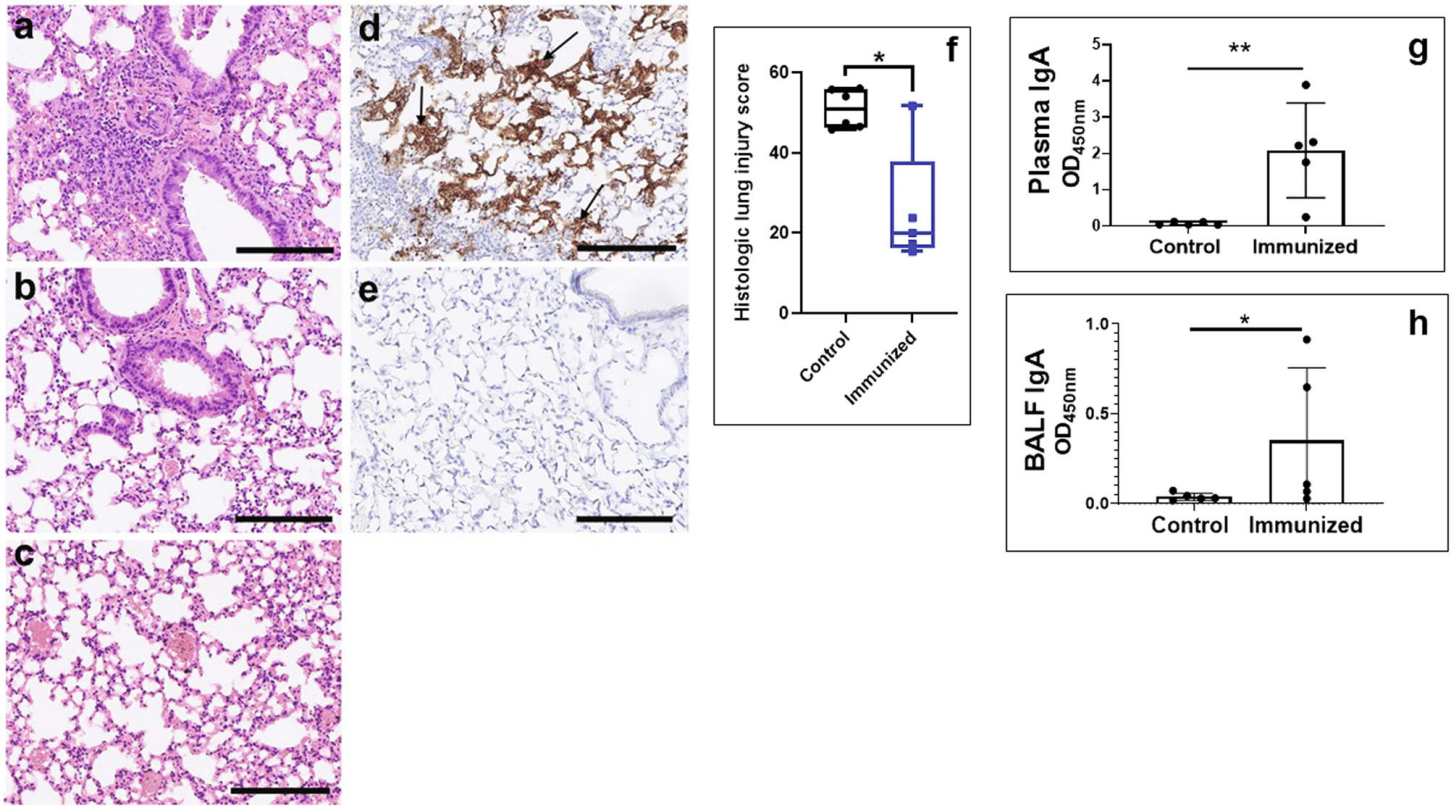
Two immunizations were sufficient to protect mice from lung pathology and elicited a robust IgA response. K18-hACE2 mice were immunized with adjuvanted Spike vaccine using a subcutaneous prime (day-0) and intranasal boost (day-21). Mice were challenged 2-weeks postsecond immunization and lungs were sampled on the day of the maximal clinical score (day-6) requiring animal euthanasia in the control group, an equal number of vaccinated mice were also euthanized. Representative H & E stained histology sections: Control mice (**a**) showed severe lung injury with marked infiltration of immune cells whereas immunized mice (**b**) showed attenuated lung injury closer to lung histology in a normal mouse (**c**). Immunohistochemistry: Lungs were stained with SARS-CoV-2 antinucleocapsid antibodies. Control mice (**d**) showed presence of virus (arrows) whereas vaccinated mice (**e**) showed clear lungs. Lung injury score. Histology sections were scored in a blinded manner (**f**). IgA ELISAs: Plasma (**g**) and bronchoalveolar lavage fluid (**h**) samples were harvested a week after the second immunization and anti-S1 response was tested using ELISA. Boxes denote mean with standard deviation. Scale bar = 100 μm. Asterisks indicate that the results are statistically significant as follows: *p < 0.05; **p < 0.01.

To determine whether two immunizations were sufficient to protect mice from lung immunopathology, K18-hACE2 mice (*n* = 10 per group) were immunized using a subcutaneous prime and intranasal boost. A three-week interval was maintained between two immunizations to mimic the current immunization regimen for the commercially approved COVID-19 vaccines. Mice were challenged two weeks postsecond immunization and the experiment was terminated on day-8 postchallenge. At this time point, vaccinated mice did not exhibit any clinical signs upon challenge (mean clinical score 0.7 ± 0.5), in contrast to the control mice, which showed higher clinical scores (mean clinical score 3.7 ± 1.9, *p* = 0.002). While six of the 10 mice in the control group succumbed to infection by day 8, all the vaccinated mice survived. Lungs obtained from the control mice during peak infection revealed multifocal inflammatory infiltrates (Fig. 2a) whereas vaccinated mice showed significantly reduced lung injury (Fig. 2b) more similar to uninfected mice (Fig. 2c). Control mice also revealed significantly higher viral load as seen after staining with antinucleocapsid antibodies (Fig. 2d) as compared to vaccinated mice (Fig. 2e). Histologic injury scoring revealed significantly higher scores for the control mice versus the vaccinated mice (Fig. 2f). Finally, we evaluated the ability of the experimental vaccine to elicit local and systemic SARS-CoV-2 S-specific IgA response in the two immunization regimen. Robust IgA levels were detected in plasma (Fig. 2g) and bronchoalveolar lavage (BAL) fluid (Fig. 2h) in the mice vaccinated using subcutaneous prime- intranasal boost regimen, whereas control mice failed to show any anti-Spike IgA response.

## Discussion

The most important outcome of this work was a demonstration of the rapid development of a highly efficacious COVID-19 vaccine using a dual TLR ligand liposome adjuvant. This adjuvant platform was recently characterized for its ability to concurrently develop a strong mucosal, as well as a Th1 type systemic immune response using amebiasis vaccine as a model^13^. Adjuvanted spike vaccine-induced robust systemic neutralizing antibodies and completely protected mice from a lethal SARS-CoV-2 challenge. The lung histology from vaccinated mice showed relative protection from the robust lung injury seen in the control mice. These observations were also reflected in the lung viral loads. Thus, this fully synthetic liposomal adjuvant administered with SARS-CoV-2 spike antigen showed an excellent protection efficacy in a mouse model of COVID-19.

COVID-19 patients show highly variable plasma neutralizing antibody response^16^ and data about the durability of neutralizing antibodies is unclear^17,18^. An effective vaccine should be able to elicit high levels of neutralizing antibodies to the SARS-CoV-2 spike protein and TLR based adjuvants have been shown to enhance effective neutralizing antibody titers against many viruses^19,20^. The inclusion of TLR ligands in adjuvanted subunit COVID vaccine elicited a Th1 biased response in monkeys compared to those without TLR ligands in a recent study and a Th1 biased response may be beneficial against COVID-19^10,21^. Our adjuvant platform elicited a robust anti-Spike neutralizing antibody response which was comparable to that seen in convalescent plasma from COVID-19 patients.

Another important observation was the demonstration that two doses were sufficient to prevent lung injury in vaccinated mice. Control mice consistently showed significantly higher clinical scores compared to vaccinated mice. Histologically, vaccinated mice showed reduced infiltration of immune cells into the alveolar interstitium throughout the course of the experiment and no detectable virus upon staining with virus-specific antibodies. Control mice, on the other hand, consistently showed lung infiltration of immune cells by histology, as well as high viral burden by immunohistochemistry.

Coordinated local mucosal and systemic immune responses are important for protection against COVID-19 pathologies. An ideal COVID-19 vaccine should be able to generate a strong mucosal as well as systemic response in both the humoral and cellular immune compartments. Immunization in this study elicited an anti-Spike IgA response in plasma as well as BAL fluid, confirming the potential of the current adjuvant combination to elicit antigen-specific mucosal IgA response^13^. Several recent studies have emphasized the importance of SARS-CoV-2 specific IgA antibodies, especially within the respiratory system, in providing effective immunity^22^. Specifically, dimeric IgA which is a secretory form at mucosal surfaces was 15-fold more potent than their monomeric counterparts^23^. Sterlin et al. showed that IgA contributed to virus neutralization to a greater extent compared with IgG in an infected cohort of patients^24^. Moreover, although IgA serum concentrations decreased one month after the onset of symptoms, neutralizing mucosal IgA remained detectable in saliva for a longer time. Thus, mucosal anti-Spike IgA response appears to be a critical feature of an effective vaccine as it can play a crucial role in barrier function at the first port of viral entry. GLA 3M-052 liposomes were efficient in generating antigen-specific mucosal IgA response, especially when used via mucosal regimen^13^ and this might be one of the reasons for 100% protective efficacy in the current study.

Our preliminary data indicate that although subcutaneous prime- intranasal booster combination elicited local and systemic IgA response, interestingly, splenocytes from these mice did not produce detectable IFN-γ upon re-stimulation with the antigen (Supplementary Fig. 1). We have not checked IFN- γ levels in the BAL fluid. It is known that intranasal vaccination is capable of stimulating immune responses in the nasopharynx-associated lymphoid tissue and the respiratory tract in addition to systemic locations^25^. Intranasal immunization with RBD domain of the S protein in MERS-CoV was shown to elicit a more powerful local mucosal immune system in the lung tissue in comparison with the subcutaneous immunization^26^. This also supports our previous observations that intranasal immunization helps induce a strong mucosal immune response compared to parenteral routes. This intriguing observation needs further characterization.

There are several limitations to this study. We have not assessed the distribution and magnitude of immune response elicited by parenteral or intranasal regimens by themselves or their combinations and it will be difficult at this point to correlate any particular regimen with protection. Additionally, antigen and excipient doses, the interval between immunizations and gender effect need to be deciphered. Both local and systemic cellular immune responses also need to be characterized. One of the key knowledge gaps is uncertainty about the longevity of response for SARS-CoV-2. Although the adjuvant was capable of generating a sustained antibody and T cell response over several months against an amebiasis antigen^15^, a Spike-specific memory response needs to be tested. A second important knowledge gap is whether cross protection will be observed against emerging mutants. In addition, control groups including antigen alone and antigen plus single adjuvant are not included and therefore one cannot truly determine the value of the adjuvant(s) in the enhancement of the immune responses and protection generated.

Together, these data demonstrated that this synthetic adjuvant system with pharmacologically acceptable components elicited a robust antigen-specific humoral response against an RNA virus and fully protected mice from clinical disease. While we have not characterized cellular immune responses, our previous studies indicated a strong antigen-specific T cell response when using GLA 3M-052 liposomes. Development of new COVID-19 vaccines with equivalent efficacies to currently approved vaccines remains essential with special focus on cross protection, the durability of the immune response, ease of administration, stability, as well as cost. For the present and future, emerging or re-emerging pandemic diseases, there will be an overwhelming demand for cost-effective vaccines, which can be developed rapidly. The availability of an adjuvant that has been tested in pre-clinical and clinical trials can address most of these requirements. Overall, GLA 3M-052 Liposome is a promising “plug-and-play” adjuvant platform for the rapid development of effective and safe vaccines.

## Methods

### Expression and purification of SARS-CoV-2 spike protein

The SARS-CoV-2 prefusion spike ectodomain protein was recombinantly expressed in mammalian ExpiCHO-S^TM^ cells (ThermoFisher, Waltham, MA) using a gene encoding 1–1281 residues, modified from GenBank: MN908947 (severe acute respiratory syndrome coronavirus 2 isolate Wuhan-Hu-1, complete genome)^27,28^. Basically, the construct contains an N-terminal signal peptide derived from µ-phosphatase, a “SGAG” substitution at the furin cleavage site (residues 701–704), proline substitutions at residues 1005 and 1006, and a C-terminal extension that contains a TEV cleavage site, a C-terminal T4 fibritin trimerization motif and an 8XHisTag. The signal peptide and amino acid sequence are identical to those in Chain A (PDB code: 6VYB_A) of the SARS-CoV-2 spike ectodomain structure in the open state. Cryo-EM studies have demonstrated that this ectodomain undergoes trimerization^27^ The DNA was synthesized by GeneArt (Regensburg, Germany) with codon optimization for expression in CHO cells and cloned into the mammalian expression vector pcDNA3.4 (Thermo Fisher Scientific, Waltham, MA, USA). The plasmid was quantified by UV spectroscopy and the sequence was confirmed by Eurofins Genomics (Louisville, KY, USA). Transfection-grade plasmids were prepared using PureLink MidiPrep kit (Thermo Fisher Scientific, Waltham, MA, USA). Plasmids were sterilized by filtration through a 0.2 µm spin filter (Corning, Tewksbury, MA, USA). Plasmid DNA was combined with ExpiFectimine^TM^ (Thermo Fisher, Waltham, MA) and transfected into ExpiCHO-S^TM^ cells (Thermo Fisher, Waltham, MA). Post-transfection, cells were cultured continuously for 13 days at 37 °C, 8% CO_2_ for secretion of the spike protein into the cell supernatant. The cell supernatant was collected, centrifuged at 2000x *g* for 30 min, and filtered through a 0.2 µm vacuum filter (Corning, Tewksbury, MA, USA). Recombinant SARS-CoV-2 spike protein expressed by CHO cells was purified using an AKTA Pure system (Cytivia, Marlborough, MA, USA). Talon resin (Cytivia, Marlborough, MA, USA) was washed with 20 column volumes of 50 mM sodium phosphate, 300 mM NaCl, 5 mM imidazole, pH 7.4. Elution was done with 50 mM sodium phosphate, 300 mM NaCl, 150 mM imidazole, pH 7.4 in 2 mL fractions using a fraction collector. Fractions containing detectable spike protein, by measuring absorbance at A280 nm, were combined and dialyzed into PBS using a Float-A-Lyzer G2 dialysis device (Repligen Corporation, Rancho Dominguez, CA, USA). The recombinant SARS-CoV-2 spike protein was soluble in phosphate-buffered saline (PBS). The purity of the SARS-CoV-2 spike protein was assessed by silver-stained SDS-PAGE (Supplementary Fig. 2) and mass spectrometry (>95% of unique peptide signal intensity). Purified spike protein was prepared for LC/MS analysis by trypsin digestion. Peptide measurements were performed using ddMS^2 data acquisition on a Q Exactive Hybrid Quadrupole Orbitrap combined with a Vanquish Flex UHPLC system (Thermo Fisher, Waltham, MA, USA). Peptide mapping of spectra was performed with Sequest HT node using Proteome Discover 2.2 software (Thermo Fisher, Waltham, MA). Endotoxin levels were assessed by Endosafe Limulus Amebocyte Lysate Assay (Charles River Laboratories Worcester, Massachusetts).

### Adjuvant formulation

GLA (Avanti Polar Lipids), 3M-052 (3 M Company), dipalmitoylphosphatidylcholine (DPPC, Lipoid), PEGylated dipalmitoyl phosphatidylethanolamine (mPEG2000-DPPE, Corden), cholesterol (Sigma), and α-tocopherol (Spectrum Chemical) were mixed in chloroform. Following evaporation of the organic solvent by overnight rotary evaporation, the lipid film was hydrated with 25 mM ammonium phosphate buffer, pH 5.8, to achieve concentrations of 1 mg/ml GLA, 0.4 mg/ml 3M-052, 3.4 mg/ml DPPC, 1 mg/ml mPEG2000-DPPE, 1 mg/ml cholesterol, and 0.09 mg/ml α-tocopherol. The mixture was sonicated for ~1 h in a water bath at 60 °C. The formulation was then processed at 18,000 psi for 5 cycles on a model LM20 microlfuidizer (Microfluidics Corp.), followed by filtration through a 0.8/0.2-µm membrane and storage in glass vials at 2–8 °C. The adjuvant formulation was mixed at 1:1 volume with antigen and saline diluent at the time of immunization, resulting in an administered adjuvant dose of 10 µg GLA and 4 µg 3M-052.

### Immunoassay to assess antibody binding to the spike S1 domain

A flat bottom 96-well clear polystyrene plate (Nunc, Rochester, New York) was coated with rSARS-CoV-2 S1 domain (Wuhan Hu-1, amino acids 14 to 685, GeneScript, New Jersey) at 5 µg/ml in coating buffer (50 mM Carbonate/Bicarbonate pH 9.6) and incubated overnight at 4 °C. The wells were washed three times with PBS-0.1% Tween 20 (PBS-T) and blocked with 100 µl/well of PBS-T containing 1% bovine serum albumin (PBS-T 1% BSA) by incubating for 1 h at room temperature. Dilutions of the test (immunized) and control (nonimmunized) mice plasma were prepared in PBS-T 1% BSA. As a standard, immune plasma was combined in a pool. After washing the blocked plate three times with PBS-T, each plasma dilution was dispensed at 100 µl/well. After incubation for 1 h at room temperature, the plate was washed again as above. The secondary antibody, peroxidase-conjugated affinity-purified goat antimouse IgG F(ab’)_2_ (Catalogue # 115-036-006, Jackson ImmunoResearch Labs, West Grove, PA), was prepared at 1: 2000 dilution in PBS-T 1% BSA, dispensed at 100 µl/well, and incubated at room temperature for 1 h. The plate was then washed three times with PBS-T, and the ABTS (2,2’-Azinobis [3-ethylbenzothiazoline-6-sulfonic acid]-diammonium salt) substrate (1 mM ABTS in 70 mM citrate phosphate buffer, pH 4.2, containing 0.1% hydrogen peroxide) was dispensed at 100 µl/well. The plate was incubated at room temperature until the green color was developed and the absorbance of the lowest dilutions (1:100) at 405 nM reached between 2.0 and 2.5. The absorbance at each well was then read at 405 nm. For IgA ELISAs, a twofold diluted plasma or undiluted BAL fluid samples were used to check for the presence of anti-S1 antibodies. Antimouse IgA-HRP (Catalogue # 1040-05, SouthernBiotech) at 1:5000 dilution prepared in PBS-T 1% BSA was used as a secondary antibody and absorbance was read at 450 nm after addition of TMB peroxidase substrate (Seracare).

### Immunizations

K18 hACE-2 transgenic C57/B6 male mice were purchased from the Jackson Laboratories. Six to ten weeks old mice were immunized subcutaneously (neck region) or intranasally under anesthesia with either 2-weeks interval (three immunization regimen) or 3-weeks interval (two immunization regimen) as mentioned in the figure legends. Typically, each mouse received 10 μg antigen that was mixed with adjuvants (10 µg GLA and 4 µg 3M-052 in liposomes) just prior to immunization. An intranasal dose consisted of 10 μl per nare whereas the subcutaneous dose was 100 μl in the neck region per animal. A 2-week interval was based on our previous studies using these adjuvants. A 3-week interval in the second experiment was based on the actual interval recommended in humans for commercial vaccines. Regimen selection was based upon our work on the amebiasis vaccine^13^. For the immunogenicity experiments, tissues were harvested from a subset of control and vaccinated mice a week after the final immunization.

### Viral propagation

SARS-CoV-2 isolate Hong Kong/VM20001061/2020 (NR-52282), was obtained from the Biodefense and Emerging Infections Research Resources Repository (BEI Resources), National Institute of Allergy and Infectious Diseases (NIAID), National Institutes of Health (NIH). Virus was propagated in Vero C1008, Clone E6 (ATCC CRL-1586) cells cultured in Dulbecco’s Modified Eagle’s Medium (DMEM, Gibco 11995040) supplemented with 10% fetal bovine serum (FBS) and grown at 37 °C, 5% CO_2_.

### Challenge

K18 hACE-2 transgenic mice were immunized either two or three times as mentioned in the figure legends. Mice were challenged intranasally 2 weeks after final immunization under ketamine/xylazine anesthesia with 1300 Plaque Forming Units (PFUs) of SARS-CoV-2 (Passage-2) in 50 μl. Mice were observed daily for clinical signs. Categories included in clinical scoring included weight loss, posture and appearance of fur (piloerection), activity, and eye closure. All mouse work was approved by the University’s Institutional Animal Care and Use Committee, and all procedures were performed in the university’s certified animal Biosafety Level three laboratory.

### IFN-γ measurement

Splenocytes were counted after RBC lysis and a total of 2 × 10^5^ splenocytes/well in 200 μl were stimulated with Spike antigen at 20 μg/ml or left unstimulated in a 96 well U-bottom plate for 72 h at 37 °C, 5% CO_2_. Supernatants were banked at −20 °C. Supernatants were analyzed for secreted IFN-γ by cytokine bead array according to the manufacturer’s instructions (Bio-Techne). The signal from splenocytes stimulated with PMA-ionomycin served as a positive control and the signal from blank well-containing medium alone served as a negative control.

### Histology

Tissues were fixed in formalin. Slides were scanned at 20x magnification. Hematoxylin and eosin (H&E) stained histology sections were scored in a blinded manner using the guidelines of the American Thoracic Society^29^ and included the following parameters-neutrophils in the alveolar space, neutrophils in the interstitial space, hyaline membranes, proteinaceous debris filling the air spaces, and alveolar septal thickening. Lungs from age-matched mice served as a negative control.

### Immunohistochemistry

Slides of fixed lung tissue were stained with SARS-CoV-2 specific anti-nucleoprotein antibody (Catalogue # 9099, ProSci, Poway, CA) as per manufacturer’s instructions at the University of Virginia’s biorepository and tissue research core facility. Slides were scanned at 20x magnification.

### Pseudovirus neutralization assay

Antibody potency was assessed by standard pseudovirus neutralization assays, in which the concentration of antiserum required to prevent cell infection by nonreplicative pseudotyped SARS-CoV-2 virus is determined. Assays were performed as described follows^30^. Briefly, pseudovirus was generated using a plasmid encoding SARS-CoV-2 S protein (Wuhan-Hu-1) with the 19 C-terminal amino acids removed, a MLV Gag-Pol helper plasmid, and a plasmid encoding a luciferase gene containing a packaging sequence (the latter two gifts of Judith White, UVA). Approximately 1 million HEK 293 T/17 cells were transfected with 1 µg plasmid and 3 µL Lipofectamine 2000. The supernatant containing pseudovirus particles was collected after 48 h, clarified for cell debris at 700 x *g* for 7 min at 4 °C, filtered through a 0.45 µm PES syringe filter, stored at 4 °C, and used within 24 h.

Infection with pseudovirus causes luciferase expression and was quantitatively assayed via luminescence 72 h after infection, via which IC_50_ values for antibody inhibition of cellular infection were calculated. Plasma samples were diluted to the specified ratio, and combined with a standardized amount of pseudovirus, and incubated for 1 h at 37 °C to permit antibody binding before addition to approximately 125,000 Vero E6 cells per well of a 24-well plate. Plates were centrifuged for 30 min at 100 x *g*, 4 °C for pseudovirus attachment and then incubated at 37 °C, 5% CO2. After 6 h, 400 µl complete media was added to each well. Luciferase activity was measured in a plate reader 60–78 h postinfection using the BriteLite reagent (PerkinElmer). Uncertainty quantification was performed via bootstrap resampling. Discarded plasma sample collected on day-32 post-COVID-19 detection from University of Virginia medical center inpatient was used as a source of convalescent plasma (IRB-HSR #22231 and 200110).

### Statistics

GraphPad Prism, Matlab and Microsoft excel were used to generate figures. A two-tailed Student’s *t* test was used to determine statistical significance for IgG ELISAs. Mann-Whitney test was used to calculate statistical significance between the groups for IgA ELISAs and clinical scores. Log-rank Mantel-Cox test was used to determined statistical significance for survival analysis. Histological scoring was analyzed using a two-tailed Student’s *t* test.

### Reporting Summary

Further information on research design is available in the Nature Research Reporting Summary linked to this article.

## Supplementary information

Supplementary information

Reporting Summary

## Data Availability

The authors declare that the main data supporting the findings of this study are available within the article and its supplementary information files. Extra data are available from the corresponding author upon request.
