# Supplementary material for: Development of COVID-19 vaccine using a dual Toll-like receptor ligand liposome adjuvant
Source: NPJ Vaccines. 2021 Nov 18;6:137. doi: 10.1038/s41541-021-00399-0 (PMC8602664; doi:10.1038/s41541-021-00399-0)
Supplement: Supplementary file 1 — Supplementary information [file 41541_2021_399_MOESM1_ESM.pdf]

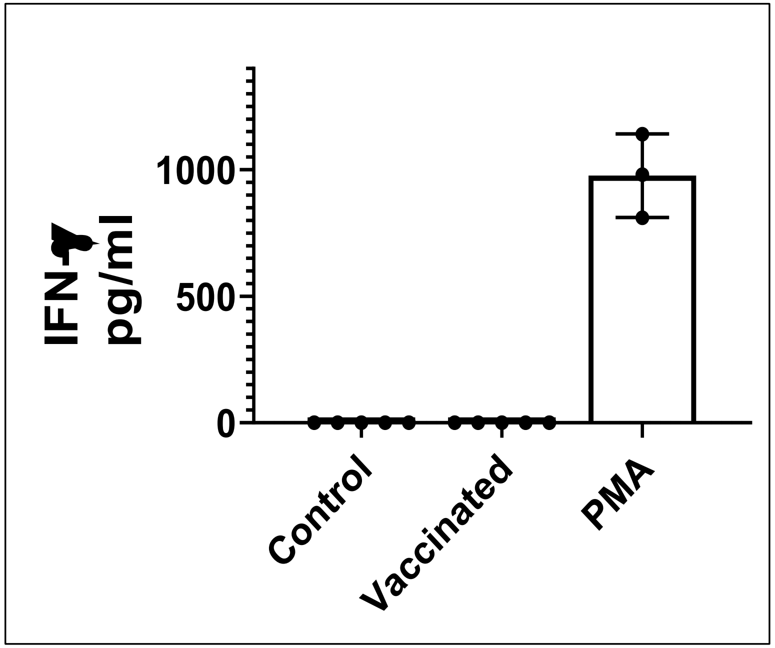

Supplementary Figure 1: **IFN-γ response.** K18-hACE2 mice were immunized with adjuvanted Spike vaccine using a two dose regimen consisting of subcutaneous prime (day-0) and intranasal boost (day-21). Samples were collected a week after final immunization. Splenocytes were re-stimulated with 10μg/ml Spike antigen for 72h. IFN-γ was measured in culture supernatant using cytokine bead assay. Box denotes mean with standard deviation. PMA: Phorbol Myristate Acetate stimulation as a positive control.

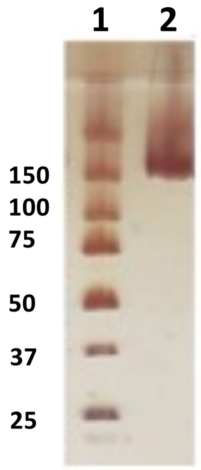

Supplementary Figure 2 : **Spike purification**: Purified full length spike protein was run on SDS-PAGE gel and silver stained.
Lane 1- molecular weight marker
Lane 2- purified full length spike
